# 1,2-Bis(4-meth­oxy­phen­oxy)ethane

**DOI:** 10.1107/S1600536813007009

**Published:** 2013-03-16

**Authors:** Jun Ji, XiuQin Zhang, Kai Wang, ChaoFan Ju, Qiang Chen

**Affiliations:** a School of Petrochemical Engineering, Changzhou University, Changzhou 213164, Jiangsu, People’s Republic of China; b High Technology Research Institute of Nanjing University, Changzhou 213162, Jiangsu, People’s Republic of China

## Abstract

The whole mol­ecule of the title compound, C_16_H_18_O_4_, is generated by twofold rotational symmetry; the twofold axis bis­ects the central C—C bond. The O—C—C—O torsion angle about the central C—C bond is 69.45 (16)°. Symmetry-related benzene rings are inclined to one another by 64.91 (8)°. In the crystal, mol­ecules are connected by C—H⋯O hydrogen bonds, forming a three-dimensional network.

## Related literature

For the synthesis, uses and properties of the title compound, see: Saito *et al.* (1988 ▶). For bond-length data, see: Allen *et al.* (1987 ▶).
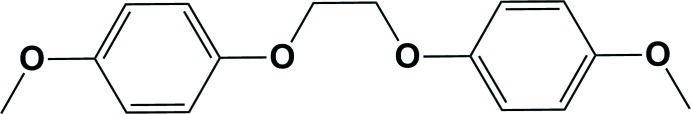


## Experimental

### 

#### Crystal data

C_16_H_18_O_4_
*M* *_r_* = 274.32
Monoclinic, 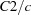

*a* = 26.073 (4) Å
*b* = 5.5538 (8) Å
*c* = 9.7591 (14) Å
β = 102.211 (3)°
*V* = 1381.2 (4) Å^3^
*Z* = 4
Mo *K*α radiation
μ = 0.09 mm^−1^
*T* = 293 K
0.20 × 0.18 × 0.15 mm

#### Data collection

Enraf–Nonius CAD-4 diffractometer
Absorption correction: ψ scan (North *et al.*, 1968 ▶) *T*_min_ = 0.981, *T*_max_ = 0.986
3780 measured reflections
1277 independent reflections
976 reflections with *I* > 2σ(*I*)
*R*_int_ = 0.023
3 standard reflections every 200 reflections intensity decay: 1%

#### Refinement

*R*[*F* ^2^ > 2σ(*F* ^2^)] = 0.046
*wR*(*F* ^2^) = 0.224
*S* = 1.10
1277 reflections
93 parameters
H-atom parameters constrained
Δρ_max_ = 0.26 e Å^−3^
Δρ_min_ = −0.29 e Å^−3^

### 

Data collection: *CAD-4 Software* (Enraf–Nonius, 1985 ▶); cell refinement: *CAD-4 Software*; data reduction: *XCAD4* (Harms & Wocadlo,1995 ▶); program(s) used to solve structure: *SHELXS97* (Sheldrick, 2008 ▶); program(s) used to refine structure: *SHELXL97* (Sheldrick, 2008 ▶); molecular graphics: *SHELXTL* (Sheldrick, 2008 ▶) and *Mercury* (Macrae *et al.*, 2008 ▶); software used to prepare material for publication: *SHELXTL*.

## Supplementary Material

Crystal structure: contains datablock(s) I, gobal. DOI: 10.1107/S1600536813007009/su2572sup1.cif

Structure factors: contains datablock(s) I. DOI: 10.1107/S1600536813007009/su2572Isup2.hkl

Supplementary material file. DOI: 10.1107/S1600536813007009/su2572Isup3.cml

Additional supplementary materials:  crystallographic information; 3D view; checkCIF report

## Figures and Tables

**Table 1:** Hydrogen-bond geometry (Å, °)

*D*—H⋯*A*	*D*—H	H⋯*A*	*D*⋯*A*	*D*—H⋯*A*
C1—H1*B*⋯O1^i^	0.96	2.60	3.453 (2)	148
C6—H6⋯O2^ii^	0.93	2.59	3.490 (2)	163

Symmetry codes: (i) 
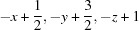
; (ii) 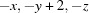.

**Fig. 1.**
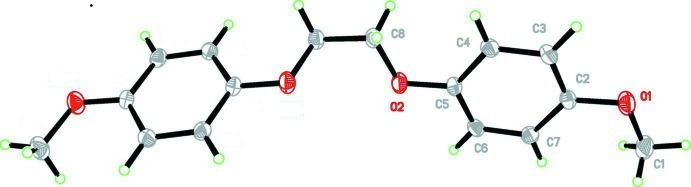
The molecular structure of the title molecule, with the atom labelling. Displacement ellipsoids are drawn at the 50% probability level.

**Fig. 2.**
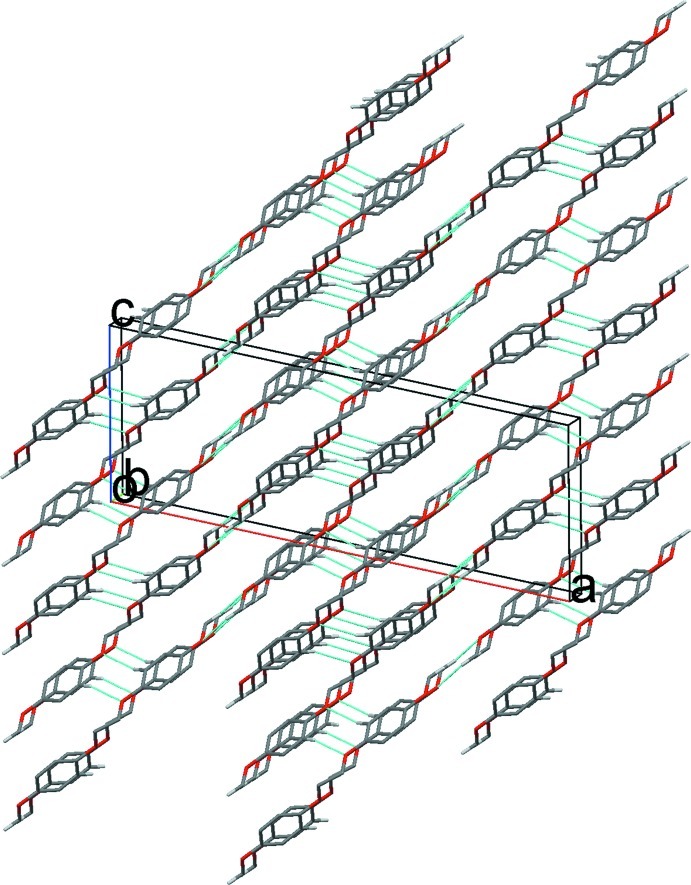
A view along the b axis of the crystal packing of the title compound, showing the C-H···O hydrogen bonds (dashed lines; see Table 1 for details; H atoms not involved in these interactions have been omitted for clarity).

## supplementary crystallographic information

### Comment

The tittle compound is used as an important organic synthesis intermediate. It is used as a sensitizer for thermal recording materials and materials for polyester-resin monomers and fire-resistant materials (Saito *et al.*,1988). We report herein on the crystal structure of the title compound.

The molecular structure of the title molecule is shown in Fig. 1. The bond lengths (Allen *et al.*, 1987) and angles are within normal ranges. The molecule has two-fold rotational symmetry, the 2-fold axis bisecting bond C8-C8A. The central O2-C8-C8A-O2A torsion angles is 69.45 (16) °. The symmetry related benzene rings [C2-C7 and C2A-C7A; symmetry code (A) -x, y, -z-1/2], are inclined to one another by 64.91 (8) °.

In the crystal, molecules are linked via C—H···O hydrogen bonds forming a three-dimensional network (Fig. 2 and Table 1).

### Experimental

The title compound was prepared by a method reported in literature (Saito *et al.*, 1988). To a solution of 1,2-dibromoethane (2.3 g, 12 mmol) and 4-methoxyphenol (3.1 g, 25 mmol) in acetonitrile (100 ml) was added anhydrous potassium carbonate (6.2 g, 45 mmol), and the mixture was stirred overnight at 338 K. The reaction mixture was filtered and the filtrate evaporated under reduced pressure. The residue was subjected to flash chromatography on silica gel, eluting with (10:1/ petroleum ether:ethyl acetate) to give title compound (Yield 2.13 g). Colourless block-like crystals of the title compound were obtained by slow evaporation of a solution in ethanol (20 ml) after about 7 d.

### Refinement

All H atoms were positioned geometrically and constrained to ride on their parent atom: C—H = 0.93 Å (aromatic H), 0.97 Å (CH_2_), and 0.96 Å (CH_3_) with U_iso_(H) = k × U_eq_(C), where k = 1.5 for CH_3_ H atoms, and = 1.2 for other H atoms.

### Crystal data

**Table:**

C_16_H_18_O_4_	*F*(000) = 584
*M**_r_* = 274.32	*D*_x_ = 1.319 Mg m^−^^3^
Monoclinic, *C*2/*c*	Mo *K*α radiation, λ = 0.71073 Å
Hall symbol: -C 2yc	Cell parameters from 1571 reflections
*a* = 26.073 (4) Å	θ = 3.2–30.3°
*b* = 5.5538 (8) Å	µ = 0.09 mm^−^^1^
*c* = 9.7591 (14) Å	*T* = 293 K
β = 102.211 (3)°	Block, colourless
*V* = 1381.2 (4) Å^3^	0.20 × 0.18 × 0.15 mm
*Z* = 4	

### Data collection

**Table:**

Enraf–Nonius CAD-4 diffractometer	976 reflections with *I* > 2σ(*I*)
Radiation source: fine-focus sealed tube	*R*_int_ = 0.023
Graphite monochromator	θ_max_ = 25.5°, θ_min_ = 1.6°
ω/2θ scans	*h* = −31→24
Absorption correction: ψ scan (North *et al.*, 1968)	*k* = −6→5
*T*_min_ = 0.981, *T*_max_ = 0.986	*l* = −11→11
3780 measured reflections	3 standard reflections every 200 reflections
1277 independent reflections	intensity decay: 1%

### Refinement

**Table:**

Refinement on *F*^2^	Secondary atom site location: difference Fourier map
Least-squares matrix: full	Hydrogen site location: inferred from neighbouring sites
*R*[*F*^2^ > 2σ(*F*^2^)] = 0.046	H-atom parameters constrained
*wR*(*F*^2^) = 0.224	*w* = 1/[σ^2^(*F*_o_^2^) + (0.168*P*)^2^] where *P* = (*F*_o_^2^ + 2*F*_c_^2^)/3
*S* = 1.10	(Δ/σ)_max_ < 0.001
1277 reflections	Δρ_max_ = 0.26 e Å^−^^3^
93 parameters	Δρ_min_ = −0.29 e Å^−^^3^
0 restraints	Extinction correction: *SHELXL97* (Sheldrick, 2008), Fc^*^=kFc[1+0.001xFc^2^λ^3^/sin(2θ)]^-1/4^
Primary atom site location: structure-invariant direct methods	Extinction coefficient: 0.020 (6)

### Special details

**Table:**

Geometry. Bond distances, angles etc. have been calculated using the rounded fractional coordinates. All su's are estimated from the variances of the (full) variance-covariance matrix. The cell esds are taken into account in the estimation of distances, angles and torsion angles
Refinement. Refinement of *F*^2^ against ALL reflections. The weighted *R*-factor *wR* and goodness of fit *S* are based on *F*^2^, conventional *R*-factors *R* are based on *F*, with *F* set to zero for negative *F*^2^. The threshold expression of *F*^2^ > σ(*F*^2^) is used only for calculating *R*-factors(gt) *etc*. and is not relevant to the choice of reflections for refinement. *R*-factors based on *F*^2^ are statistically about twice as large as those based on *F*, and *R*- factors based on ALL data will be even larger.

### Fractional atomic coordinates and isotropic or equivalent isotropic displacement parameters (Å^2^)

**Table:**

	*x*	*y*	*z*	*U*_iso_*/*U*_eq_	
O1	0.20123 (6)	0.7928 (2)	0.33002 (13)	0.0496 (5)	
O2	0.02888 (5)	0.7020 (2)	−0.10672 (11)	0.0412 (5)	
C1	0.20477 (9)	0.9943 (3)	0.42124 (19)	0.0545 (8)	
C2	0.15790 (7)	0.7773 (3)	0.22314 (17)	0.0332 (6)	
C3	0.15573 (7)	0.5837 (3)	0.13368 (16)	0.0364 (6)	
C4	0.11377 (7)	0.5526 (3)	0.02195 (16)	0.0343 (6)	
C5	0.07272 (7)	0.7183 (3)	−0.00101 (16)	0.0316 (5)	
C6	0.07499 (7)	0.9113 (3)	0.08874 (15)	0.0352 (6)	
C7	0.11697 (7)	0.9435 (3)	0.20009 (16)	0.0361 (6)	
C8	0.02601 (7)	0.5040 (3)	−0.20015 (15)	0.0369 (6)	
H1A	0.20640	1.13950	0.36900	0.0820*	
H1B	0.23590	0.98070	0.49380	0.0820*	
H1C	0.17450	0.99880	0.46250	0.0820*	
H3	0.18300	0.47210	0.14870	0.0440*	
H4	0.11300	0.42150	−0.03770	0.0410*	
H6	0.04770	1.02230	0.07400	0.0420*	
H7	0.11790	1.07540	0.25930	0.0430*	
H8A	0.03060	0.35460	−0.14740	0.0440*	
H8B	0.05390	0.51580	−0.25180	0.0440*	

### Atomic displacement parameters (Å^2^)

**Table:**

	*U*^11^	*U*^22^	*U*^33^	*U*^12^	*U*^13^	*U*^23^
O1	0.0415 (9)	0.0576 (10)	0.0417 (9)	0.0100 (6)	−0.0089 (6)	−0.0100 (6)
O2	0.0360 (9)	0.0484 (9)	0.0342 (8)	0.0108 (5)	−0.0037 (6)	−0.0086 (5)
C1	0.0498 (13)	0.0594 (14)	0.0457 (12)	0.0018 (9)	−0.0094 (9)	−0.0141 (8)
C2	0.0324 (10)	0.0394 (11)	0.0261 (9)	0.0017 (7)	0.0024 (7)	0.0032 (6)
C3	0.0336 (11)	0.0408 (11)	0.0340 (10)	0.0103 (7)	0.0052 (7)	0.0028 (7)
C4	0.0373 (11)	0.0348 (10)	0.0307 (9)	0.0061 (7)	0.0067 (7)	−0.0021 (6)
C5	0.0302 (10)	0.0388 (10)	0.0250 (8)	0.0028 (6)	0.0041 (7)	0.0024 (6)
C6	0.0339 (10)	0.0355 (10)	0.0345 (10)	0.0087 (7)	0.0037 (8)	0.0003 (7)
C7	0.0393 (11)	0.0346 (10)	0.0335 (9)	0.0039 (7)	0.0054 (8)	−0.0039 (6)
C8	0.0375 (11)	0.0425 (11)	0.0286 (9)	0.0043 (6)	0.0024 (8)	−0.0033 (6)

### Geometric parameters (Å, º)

**Table:**

O1—C1	1.421 (2)	C8—C8^i^	1.493 (2)
O1—C2	1.369 (2)	C1—H1A	0.9600
O2—C5	1.371 (2)	C1—H1B	0.9600
O2—C8	1.4203 (19)	C1—H1C	0.9600
C2—C3	1.379 (2)	C3—H3	0.9300
C2—C7	1.393 (3)	C4—H4	0.9300
C3—C4	1.382 (2)	C6—H6	0.9300
C4—C5	1.393 (3)	C7—H7	0.9300
C5—C6	1.378 (2)	C8—H8A	0.9700
C6—C7	1.381 (2)	C8—H8B	0.9700
			
C1—O1—C2	117.44 (15)	H1A—C1—H1B	109.00
C5—O2—C8	117.16 (13)	H1A—C1—H1C	110.00
O1—C2—C3	116.64 (15)	H1B—C1—H1C	110.00
O1—C2—C7	124.21 (15)	C2—C3—H3	119.00
C3—C2—C7	119.16 (16)	C4—C3—H3	119.00
C2—C3—C4	121.10 (16)	C3—C4—H4	120.00
C3—C4—C5	119.81 (15)	C5—C4—H4	120.00
O2—C5—C4	124.55 (14)	C5—C6—H6	119.00
O2—C5—C6	116.53 (15)	C7—C6—H6	119.00
C4—C5—C6	118.91 (15)	C2—C7—H7	120.00
C5—C6—C7	121.42 (16)	C6—C7—H7	120.00
C2—C7—C6	119.60 (15)	O2—C8—H8A	110.00
O2—C8—C8^i^	109.61 (14)	O2—C8—H8B	110.00
O1—C1—H1A	109.00	H8A—C8—H8B	108.00
O1—C1—H1B	109.00	C8^i^—C8—H8A	110.00
O1—C1—H1C	109.00	C8^i^—C8—H8B	110.00
			
C1—O1—C2—C3	178.49 (16)	C3—C2—C7—C6	0.2 (3)
C1—O1—C2—C7	−1.2 (2)	C2—C3—C4—C5	−0.3 (3)
C8—O2—C5—C4	−1.2 (2)	C3—C4—C5—O2	−178.66 (16)
C8—O2—C5—C6	179.96 (15)	C3—C4—C5—C6	0.2 (3)
C5—O2—C8—C8^i^	175.16 (13)	O2—C5—C6—C7	179.05 (15)
O1—C2—C3—C4	−179.60 (16)	C4—C5—C6—C7	0.1 (2)
C7—C2—C3—C4	0.1 (3)	C5—C6—C7—C2	−0.3 (3)
O1—C2—C7—C6	179.87 (16)	O2—C8—C8^i^—O2^i^	69.45 (16)

Symmetry code: (i) −*x*, *y*, −*z*−1/2.

### Hydrogen-bond geometry (Å, º)

**Table:**

*D*—H···*A*	*D*—H	H···*A*	*D*···*A*	*D*—H···*A*
C1—H1*B*···O1^ii^	0.96	2.60	3.453 (2)	148
C6—H6···O2^iii^	0.93	2.59	3.490 (2)	163

Symmetry codes: (ii) −*x*+1/2, −*y*+3/2, −*z*+1; (iii) −*x*, −*y*+2, −*z*.
